# Epigenetic Regulation of Autophagy: A Path to the Control of Autoimmunity

**DOI:** 10.3389/fimmu.2018.01864

**Published:** 2018-08-14

**Authors:** Jessica C. Hargarten, Peter R. Williamson

**Affiliations:** Laboratory of Clinical Immunology and Microbiology (LCIM), National Institute of Allergy and Infectious Diseases (NIAID), National Institutes of Health (NIH), Bethesda, MD, United States

**Keywords:** histone, epigenetics, autoimmunity, autophagy, mRNA degradation, histone deacetylase inhibitors, miRNA

## Abstract

Autoimmune diseases are a significant cause of debilitation and mortality globally and are in need of cost-effective therapeutics. Autophagy is a cellular pathway that facilitates immune modulation involved in both pathogen control and autoimmunity. Regulation is multifactorial and includes a number of epigenetic pathways which can involve modification of DNA-binding histones to induce autophagy-related mRNA synthesis or microRNA and decapping-associated mRNA degradation which results in autophagy suppression. Appreciation of epigenetic-based pathways involved in autophagy and autoimmunity may facilitate application of a burgeoning group of epigenetic pharmaceuticals to these important diseases.

## Introduction

Autoimmunity-related diseases are a common cause of debilitation globally. The search for treatments is an active area of research, in no part due to the fact that three of the top six best-selling prescription drugs in 2015 were for the control of autoimmune disorders. In addition to the widespread prevalence of these diseases, the compelling economic benefit of these agents is borne out by a recent study showing that maintenance of even a mild degree of inflammation in patients resulted in comparative employee productivity to that of unaffected employees (1).

FDA approved epigenetic drugs include the histone deacetylase inhibitors romidepsin (cutaneous and peripheral T-cell lymphomas), belonostat (refractory peripheral T-cell lymphoma), panobinostat (refractory multiple myeloma), and vorinostat (refractory T-cell lymphoma) as well as a number of histone acetyltransferase inhibitors such as azacitidine and decitabine (both for chronic myelomonocytic leukemia and myelodysplastic syndrome) (2). As development progresses, it is likely that pharmaceutical epigenetic therapies will be adapted to other diseases including autoinflammatory diseases and small molecule inhibitors, such as these, may prove cost-effective. While this could have tremendous implications for patients with these diseases, it is important to identify regulatory pathways inherent to epigenetic regulation including autophagy to minimize side effects that are unexpected only because of ignorance of a relevant pathway(s). “On target” treatment toxicity is common. For example, the increased risk of *Aspergillus* infections in patients taking the B-cell-directed Bruton’s tyrosine kinase (BTK) inhibitor, ibrutinib (3), due to an unexpected role of these inhibitors in a TLR9-BTK-calcineurin-nuclear factor of activated T-cells pathway in innate immunity to the fungus (4). Thus, a thorough understanding of the impact of epigenetic pathways may be key to avoiding unexpected toxicities of these agents.

## The Balance of Autophagy During Infection and Autoimmunity

**Figure 1:**
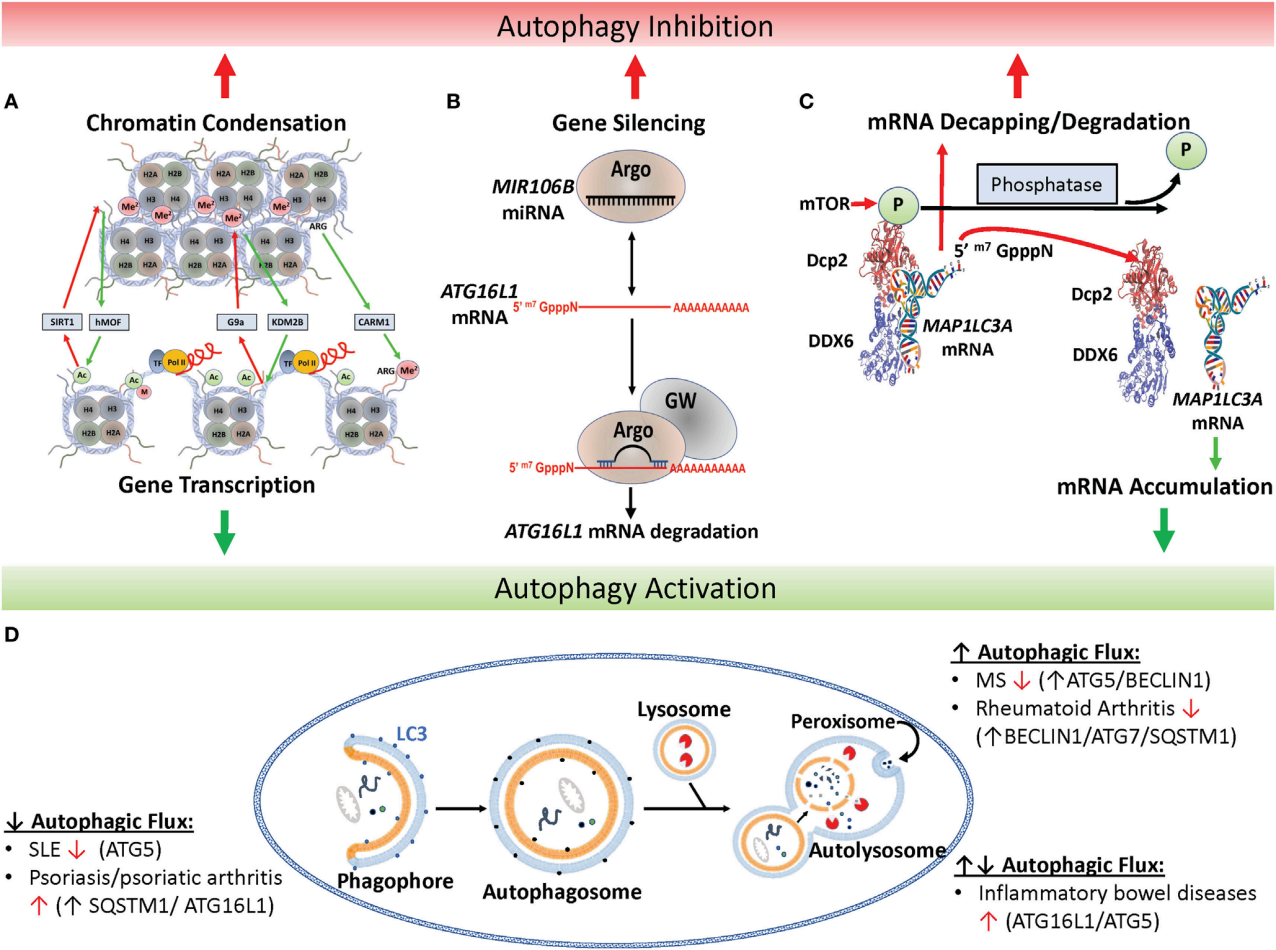
Epigenetic regulation of autophagy. **(A)** Histone marks facilitate either chromatin condensation (top panel) or an open matrix which facilitates transcription (lower panel). Repressors include the histone deacetylase SIRT1, the H3 histone methyltransferase G9a and activators include the H3 histone acetyltransferase hMof, the H3 demethylase KMS2B and the H3 arginine methyltransferase co-activator-associated arginine methyltransferase 1 (CARM1). **(B)** Canonical microRNAs (miRNAs), such as MIR106B, is recruited to Argonaut members (ARG) which recruits target mRNAs, such as the autophagy-related ATG16L1 mRNA, for degradation and gene silencing in concert with GW-motif proteins (GW). **(C)** Mechanism of mTOR-dependent decapping/degradation. mTOR-dependent phosphorylation of the decapping protein DCP2 facilitates recruitment of target mRNA molecules for decapping followed by degradation. Conversely, low mTOR activities in the presence of phosphatases result in dissociation of the decapping complex from the mRNA target with resultant accumulation of target transcripts, illustrated here with the autophagy-associated transcription, *MAP1LC3A*. [Model of putative mammalian DCP2-DDX6 interaction with *MAP1LC3A* mRNA adapted from Ref. (16).] **(D)** Illustration of autophagy and autoimmune diseases associated with alterations in autophagic flux. In autophagy, protein aggregates, misfolded proteins, and pathogens are recruited to the phagophore and then enclosed by a double-membrane vesicle to form the autophagosome. Following lysosome fusion with the autophagosome, proteinaceous material undergoes degradation in the autolysosome. Red arrow found next to autoimmune disease names indicates whether disease improves (↑) or declines (↓) following pharmacological inhibition of autophagy (17).

Autophagy was first described in yeast as a mechanism of intracellular recycling during nutrient stress (5). During cellular stress, specific autophagy-related proteins (designated Atg) orchestrate the sequestration of cytosolic materials to be recycled into a double-membraned structure, called the autophagosome (Figure 1D). Recently, the role of autophagy in mammalian immune modulation has been demonstrated in both innate and adaptive immunity (6, 7). Autophagy plays a direct role in eliminating invading pathogens by phagocytic processes (8), as well as MAP1LC3-associated phagocytosis (LAP) and sequestosome-like receptor recruitment (9). Autophagy also limits excessive inflammation during pathogen control by: removing residual microbial debris, known to activate the inflammasome pathway; digesting dysfunctional mitochondria, which typically mediate production of reactive oxygen species (ROS); or through direct removal of inflammasome complexes (10). These residual “mop up operations” of autophagy can also be induced by secondary “danger” signals (11) typically mediated by the mTOR pathway that harkens back to the role of this pathway in the yeast nutrient response. In adaptive immunity, autophagy also facilitates effective major histocompatibility complex presentation for T-cell activation (12), serving to control pathogens and remove inflammatory microbial products. Indeed, the importance of autophagy has been recognized by the pathogens themselves in that many utilize host autophagy to protect themselves against killing and support survival within host cells. The fungus *Cryptococcus neoformans*, which causes lethal meningoencephalitis (13), as well as certain bacterial pathogens, such as *Mycobacterium tuberculosis* (*Mtb*), have co-opted autophagic vesicles to conceal their intracellular residence and prevent lysosomal fusion and microbial killing (14, 15).

Although a direct link between autophagy, autoimmunity, and infectious disease is still under investigation, monogenic primary immune deficiencies in humans highlight the growing evidence for their interconnection. Activated PI3K-delta syndrome (APDS), for example, whose dysregulation results in immune-mediated cytopenias treatable by the PI3K inhibitor leniolisib (18)—has been associated with higher risk for developing autoimmune diseases (19). Similarly, chronic granulomatous disease (CGD), resulting from defects in the NADPH oxidase complex is not only associated with reduced ROS production, multiple recurrent infections (20), and chronic inflammation in patients—whose inflammatory colitis can be successfully treated with the IL-1 receptor antagonist, anakinra (21), but also autoimmunity (22). Interestingly, the increased IL-1β production in CGD was linked to a reduction in autophagy that also resulted in defects in phagocyte killing of internalized bacteria and fungi (23), demonstrating a link between autoimmunity, autophagy, and infectious disease.

Therapeutic interventions against autoimmune diseases are also strongly associated with susceptibility to infection. This is exemplified in patients undergoing treatment for multiple sclerosis (MS) who are at an increased risk of life-threatening *Histoplasma capsulatum* infections with the use of TNFα inhibitors, such as infliximab and etanercept (24), or increased risk of CNS infections with the very late antigen 4 (VLA-4) inhibitor, natalizumab, used for minimized autoimmune inflammation (25, 26). A number of “off-target” epigenetic side effects have also been described that associate autophagy and epigenetics. For example, the psychotropic drug lithium acts to downregulate HDAC1 translation, leading to decrease in histone deacetylation and upregulation of autophagy (27). Clearly, an appreciation of regulatory pathways related to autophagy and immunity will be useful to anticipate side effects of epigenetic modifying pharmaceuticals.

## mRNA Transcript Synthesis: Role of Histone Modification

The field of epigenetics has been a slowly evolving and often controversial concept in genetics. Indeed, some of the first epigenetic molecular work was published in 1964 by Allfey et al. who proposed a role for histone modifications in gene regulation. However, the field progressed slowly until ignited by the synthesis of histone epigenetic studies by Strahl and Allis (28) and Turner (29). Since that time, epigenetic studies have identified a number of covalent histone post-translational modifications, including acetylation, methylation, phosphorylation (30, 31), ADP-ribosylation (32), ubiquitination (33), SUMOylation (30), citrullination (34), glycosylation (35), hydroxylation (36), and isomerization (37, 38). Prominent among these are acetylation and methylation with a number of these histone modifications related directly to the regulation of autophagy and will therefore be the focus of this review. However, since the field of epigenetic regulation of autoimmunity is still in its infancy, many areas remain to be elucidated.

Histone post-translational modifications control gene expression by a number of mechanisms including altering the electrostatic associations between nucleosomes, modulating interactions between nucleosomes and DNA, interfering with transcription factor binding to promoter/enhancer regions, or recruiting either activating or repressing protein complexes to the specific histone modification (39). Typical modifications occur at the epsilon amino group of lysine sidechains within the polypeptide and serve to reduce the electrostatic charge of histones by acetylation and methylation or reverse this by phosphorylation. Some of the best-known modifications affecting autophagy are exhibited in Figure 1A. For example, the histone acetyltransferase hMOF/KAT8 acts to add an acetyl group to H4K16 facilitating chromatin decondensation, which sterically allows transcriptional machinery and enhancers access to DNA facilitating expression of autophagy-related genes (40). Conversely, overexpression of the NAD-dependent histone deacetylase sirtuin 1 (SIRT1) antagonizes H4K16 acetylation reducing basal levels of autophagy, which can be inhibited by the drug valproic acid (41). However, the relationship is complicated by a feedback loop whereby SIRT1 acts on non-histone targets in an mTOR-dependent fashion to induce autophagy, which subsequently inhibits hMOF/KAT8 activity (40).

SIRT1 may also play a critical role in regulating the immune system by modulating the activity of essential transcriptional regulators. Specifically, SIRT1 deacetylates RAR-related orphan receptor gamma promoting its transcriptional activity and Th17 cell differentiation (40). In thymic epithelial cells, SIRT1 is an essential regulator of AIRE-mediated expression of tissue-restricted antigens, a critical step for immunological self-tolerance (42, 43). Interestingly, polymorphisms in *SIRT1* are associated with autoimmune thyroiditis and high titers of anti-thyroid antibodies (44), suggesting a link between epigenetic regulators and autoimmunity. Immune consequences for overlapping regulation of autophagy and immunity can be seen with other related histone deacetylases. SIRT6 potentiates autophagy activation through effects on autophagy-related genes (*ATG12, ATG3*, and *ATG7*), as well as the well-known Crohn’s colitis-associated autophagy gene, *IRGM* (45, 46). Broad spectrum deacetylases, such as those found within the HDAC family—exemplified by HDAC4’s ability to deacetylate H3K9, 14, 18, and 23 and H4K5, 8, 12, and 16—are well known for their role in cancer biology prompting development of the HDAC inhibitors described above and in Table 1 (47). But they also show promise for the treatment of autoimmune diseases, as HDAC4 inhibitors have been shown to alleviate vascular inflammation resulting from activation of autophagy (48). Similarly, HDAC6 has been shown to modulate *ATG6* (BECN1) and *ATG7* and the inhibitor tubastatin A was found to potentiate autophagy with inhibition of the pro-inflammatory cytokine, IL-6 (49). These experiences suggest that further studies may identify epigenetic pathways relevant to HDAC inhibition that may prove useful in autoinflammatory disorders.

**Table 1:** Epigenetic regulators associated with autophagy and immunity.

Histone modification					
Histone modification	Regulator	Effect on autophagy	Immune phenotype	Disease implicated	Reference
H3K9Ac	SIRT6	↑ATG5	Inhibition of NOTCH/NF-κB signaling	Proteinuric kidney disease	(50–52)
H4K16Ac (H1.2 variant)	SIRT1/HDAC1	↑Autophagy	Inflammation	Diabetic retinopathy	(53)
H3K9me	HIF-1α, KDMs	↑BNIP3	Reactive oxygen species response	Traumatic brain injury/tumors	(51, 54)
H3R17me2	TFEB/co-activator-associated arginine methyltransferase 1	↑ATG14	Myeloid differentiation, SWI/SNF	Unknown	(39, 55, 56)
H4R3me2	C/EBPβ/PRMT5	Unknown	IL-8, TNFα expression	Unknown	(57)
Multiple	HDAC6	SQSTM1 autophagic clearance	Interferon response pathway	Viral/bacterial clearance	(58, 59)

**Histone deacetylase inhibitors (HDACi)**					

**Drug**	**Regulator**	**Effect on autophagy**	**Immune phenotype**	**Diseases treated with HDACi**	**Reference**

Vorinostat	HDACs	↑Autophagosome formation (ATG5)	Viral myocarditis	Cutaneous T-cell lymphoma	(60)
Vorinostat	HDACs	Unknown	CD4 and CD8 tumor immunity	Metastatic colorectal cancer	(61)
Vorinostat	HDACs	↑Autophagy (ATG5)	NF-κB signaling, VSV oncolysis	See diseases treated above	(62)
Tubastatin A	HDAC6	↑Autophagy (ATG7)	TNFα, IL-6 cisplatin toxicity	Acute kidney injury/pancreatic cancer	(49, 63)
Panobinostat	HDACs	↑Autophagy (LC3)	Lymphocyte tumor killing, TNFα	Hodgkin lymphoma/multiple myeloma	(64, 65)
Multiple	HDACs	↑Autophagic flux (ULK1/ATG7)	Reverse HIV-1 latency	Peripheral T-cell lymphoma	(66)
Multiple	HDACs	↓Autophagy (ATG7)	Apoptosis induction	DS-AMKL (proposed)	(67)

**microRNA (miRNA) regulation of autophagy**					

**miRNA**		**Effect on autophagy**	**Immune phenotype**	**Disease implicated**	**Reference**

miR-30a		↓BECN1 (↓autophagy)	Unknown	Cancer	(68)
miR-30b		↓Autophagy (↓ATG12, BECN1)	Intracellular survival of *Helicobacter pylori*	Cancer	(69, 70)
miR-106b, miR-93		↓Autophagy (↓ATG16L1)	Defects in bacterial clearance, inflammation	Crohn’s disease	(71)
miR-142-3p		↓ATG16L1	Intestinal inflammation	Crohn’s disease	(72)
miR-30c, miR-130a		↓Autophagy (↓ATG5, ATG16L1)	Invasive *Escherichia coli*, NF-κB activation, inflammation	Crohn’s disease	(73)
miR-196		↓IRGM (↓autophagy)	Mitochondrial function, ineffective *Mycobacterium tuberculosis* (*Mtb*) and *E. coli* control	Crohn’s disease	(74, 75)
miR-210		↓Bcl-2	HIF-1α pathways, hypoxia-induced apoptosis, TH_17_ differentiation	Traumatic brain injury	(76, 77)
miR-21		↓IL-12p35, ↓Bcl-2	NF-κB activation, impaired anti-mycobacterial T cell responses	*Mtb* infection, asthma	(78, 79)
miR-17, -20, -93, -106		↓SQSTM1	Elevated P-ERK levels, enhanced hematopoiesis	Acute myeloid leukemia	(80)
miR-155, -31		↓PPP2R5A (↓autophagy)	↓JAK-STAT ↑WNT-SHH, Th2 polarization	Mycobacteria, *Shigella, Listeria* infection	(81)
miR-UL148d (HCMV)		↓ERN1 (↓autophagy)	Inhibition of apoptosis, impaired anti-viral response	HCMV infection	(82)
miR-1303		↓ATG2B (↓autophagy)	Suppression of mycobacteria-induced autophagy, ↓TNF-α	*Mtb* infection	(83)
miR-471-5p		↓LC3, ↓ATG12, ↓BECN1	LC3-associated phagocytosis, apoptotic germ cells	Male infertility	(84)
miR-155		↓ATG3 (↓autophagy)	Suppression of anti-*Mtb* dendritic cell response	*Mtb* infection	(85)
miR-155		↓RHEB (↑autophagy)	Enhanced killing of intracellular *Mtb*	*Mtb* infection	(86)

A second set of histone modifiers are those involved in H3K9 methylation by the euchromatic histone-lysine N-methyltransferase 2 (G9a/EHMT2) and GLP/EHMT1 methyl transferases which form heteromeric complexes via their Su(var)3-9-Enhancer of zeste-trithorax (SET) domains (87). These lysine methyltransferases transfer one to three methyl groups from *S*-adenosyl-l-methionine to the target lysine e-amino group, causing a similar charge disruption as acetylation described above. In addition, H3K9me3 moieties (marks) recruit histone mark “readers” such as heterochromatin protein 1 (HP1) whose unique chromodomain mediates protein binding, heterochromatin formation (a tight lattice of DNA bound to histones), and transcriptional repression (Figure 1A, top panel). The methyltransferase activities of G9A/EHMT2 and the polycomb-repressive complex member, enhancer of zeste homolog 2 (EZH2), converge onto H3K27, whose methylation is associated with derepression of mTOR (88) and autophagy repression (89). Pharmacological inhibition of G9a/EHMT2 with BIX01294 results in induction of autophagy demonstrated by increased LC3B-positive autophagic vesicles (90), but autoimmunity was not studied in this context. Long non-coding RNAs (lncRNA) add an additional layer of regulation to this pathway that might hint at a connect to autoimmunity. Specifically, the lncRNA HOTAIR acts as a scaffold to recruitment the histone methyltransferase EZH2 to target genes, facilitating H3K27me-mediated gene repression (91). Aberrant expression of HOTAIR is associated with various cancers (92) and MS (93).

Interestingly, the hypoxia-inducible transcription factor (HIF1α)—recently shown to impact T-cell differentiation (94) and B-cell-related autoimmune disease (95)—was also found to regulate the H3K9 lysine (K)-specific demethylase, KDM2B, as well as the related demethylase KDM1A. This epigenetic regulation leads to activation of autophagy as well as mTOR, NF-κB, and TGF-β pathways important in T-cell adaptive signaling (96). In addition, HP1 located at H3K9me3 marked histones can recruit DNA methylases, such as DNA (cytosine-5)-methyltransferase 1 (DNMT1), which provide more permanent heterochromatin formation. DNA methylation carried out by DNMT1 have been implicated in susceptibility to endogenous retrovirus-induced systemic lupus erythematosus (97) related to increased antigen processing of hypomethylated DNA (98) or altered gene expression of inflammatory genes directly, including IL-17 (99).

An unusual histone modifying enzyme associated with autophagy is the arginine-specific H3R17 methyltransferase, co-activator-associated arginine methyltransferase 1 (CARM1), which collaborates with the transcription factor, TFEB, to enhance histone methylation allowing access of transcription factors in the AMP-SKP-CARM1 signaling cascade to autophagy-related genes (55). Previous studies have also shown a role for CARM1 as a promoter-specific regulator of NF-κB signaling (100), important for a number of innate and adaptive immune responses, demonstrating the intimate relationship between autophagy activation and autoimmunity.

## mRNA Transcript Degradation: Role of microRNAs (miRNAs)

The complexity of miRNAs has been quite daunting, but its complexity is important to the programmatic modulation of autophagy and its effect on inflammation. miRNAs are the shortest of the non-coding RNAs at approximately 22 nucleotides in length. Most miRNAs are considered “canonical” in that they undergo primary miRNA processing in the nucleus by the “microprocessor” complex, which contains the RNase III enzyme Drosha and the dsRNA-binding protein Dgcr8, and further maturation in the cytoplasm by the ribonuclease DICER. The resulting ~22 nucleotide duplex is loaded into the Argonaute-containing RNA-induced silencing complex (RISC), which then recruits mature mRNAs for degradation (Figure 1B). Interestingly, the complete structure of RISC is still unresolved and reports of size and component variability suggest complex adaptability to induction conditions or the passengers in question. In addition to “canonical” miRNAs, various non-canonical miRNAs appear to remain dependent on DICER, but are processed independent of Drosha or Dgcr8 (101). The best known of these are mirtrons, which are processed through a unique intronic splicing mechanism and have been recently associated with the pathophysiology of IgA deficiency *via* regulation of immunoglobulin heavy constant alpha 1 (*IGHA1*) and *IGHA2* (102). It is likely that this intricate web of miRNA regulation furnishes an important modulating capacity for the immune system to optimize survival against a range of pathogenic organisms under strong evolutionary pressure. But when inappropriately triggered may result in the “off-target” consequence of autoimmunity.

One of the first miRNAs identified to play a role in immunity was the global regulator miR-155. Prominently associated with IFN-γ expression and germinal center function (103–105), it was not determined until much later that some of the direct regulatory targets of miR-155 were autophagy-related (86). One of the best-known targets of miRNA-regulated autophagy is the immunity-related GTPase family M protein (IRGM) clinically associated with inflammatory bowel disease (106, 107). The canonical miRNA miR-196 targets and regulates *IRGM* whose levels confer either autophagic protection or cell death in target cells, implicated in both defense against the intracellular pathogen *Mtb* and damaging inflammation caused by Crohn’s disease. Such studies are an important demonstration of the importance of the exquisite immunological balance necessary to provide both microbiological protection and avoid autoimmune pathology. Pertinent to the importance of epigenetic mechanisms of regulation, clinical genetic studies identified a disease variant that was originally felt to be dispensable due to a lack of effects on either IRGM protein sequence or splice site selection, but later found to result in downregulation of an *IRGM* protective variant, but not a risk-associated allele due to a miRNA-based alteration in *IRGM* regulation (74).

Another prominent target of miRNA degradation is the autophagy-associated gene autophagy-related 16 like 1 (*ATG16L1*). Typically, ATG16L1 interacts with ATG12-ATG5 facilitating the phosphatidylethanolamine lipidation of the vesicular shaping protein, MAP1LC3A, and elongation of the nascent autophagosomal membrane (108, 109). In one study, levels of *MIR106B* were found to be elevated in the intestinal epithelium of patients with active Crohn’s disease along with decreased levels of *ATG16L1* transcripts (Figure 1B) (71). This was found in human cell lines to be associated with defects in autophagy-dependent eradication of intracellular bacteria. Studies in the same year identified a role for *MIR142-3p* in the same target gene (72) and others also found that Crohn’s disease-associated adherent *Escherichia coli* were able to modulate *MIR30C* and *130A* to effect changes in *ATG16L1* transcripts as well as the autophagy-conjugation gene, *ATG5* (73). Interestingly, ATG16L1 has recently been found to play a role in NOD2 inflammasome activation (110) and is associated with inflammatory bowel disease (111). Recently, *ATG16L1* gene polymorphisms were found to be associated with necrotizing enterocolitis in premature infants, again stressing the potential importance of this regulatory pathway to autoimmunity (112).

## mRNA Transcript Degradation: Role of Regulated mRNA Decapping/Degradation

A more recently identified mechanism of post-transcriptional regulation implicated a well-characterized mRNA decay pathway, characterized as a housekeeping function to remove RNA in yeast (113) and mammalian systems (114). In this process, mRNA undergoes a reversible poly-A tail deadenylation followed by an irreversible 5′-decapping by the decapping enzyme DCP2 and subsequent XRN1-exonuclease mediated degradation of the RNA in the 5′–3′ direction. More recently, studies in the yeasts *Saccharomyces cerevisiae* as well as *C. neoformans* identified an RNA-binding protein, ATP-dependent RNA helicase Dhh1/Vad1, as an RNA chaperone that binds and recruits targeted autophagy-related mRNA to the decapping complex resulting in suppression of autophagic flux (Figures 1C,D). Regulation of this process by mTOR was demonstrated by a specific mTOR-dependent phosphorylation of the DCP2 protein in humans, without which mRNA recruitment and decapping was prevented. These studies were extended to patients with monogenic dominant-activating mutations in a PI3K p110δ subunit who were further characterized by increased mTOR activity and autoimmune-associated leukopenia (115). Increased mTOR activity in these patients resulted in accelerated decapping and degradation of relevant autophagy mRNA transcripts with resultant decreased autophagy activity. Further studies, prompted by the recent finding of a role for autophagy in modulation of inflammasome-related IL-1β levels (116) demonstrated that the reduced autophagy activity in these patients resulted in elevated levels of IL-1β, suggesting an etiology of the patient’s autoimmunity. Conversely, knockdown of DDX6 by siRNA was successful in pseudonormalization of IL-1β levels, suggesting both a pathway for rapamycin-treatment of this disorder and new targets for pharmacological intervention against autophagy-related IL1β-associated autoimmunity.

## A Question of Balance and the Role of Transcriptional “Futile Cycles”

As suggested by the parallel pathways described in Figure 1, epigenetic mechanisms have the ability to modulate each pathway’s activity. This coupling of mRNA synthesis with mRNA degradation is exemplative of transcriptional “futile cycles” first describe in yeast (117). Futile cycles were first described in energy metabolism with the classic example concerning gluconeogenesis, where regulated inhibition of a degradative phosphatase resulted in a rapid accumulation of fructose 1,6-bisphosphate required for *de novo* glucose synthesis during the “fight or flight” response (118, 119). These cycles were termed futile, because it was not yet understood why energy would be exerted to simultaneously synthesize and degrade a required cellular precursor. However, maintenance of basal levels of synthesis (metabolic intermediate or mRNA) even during periods of disuse allows more rapid induction of synthetic enzymes without the need to start from zero. It also allows rapid adaptation to newly required steady states by simultaneous modulations in both synthesis and degradation. The concept is well suited for immune mechanisms, demonstrated by the induction of inflammasome activation by TLR4, accompanied by the simultaneous induction of autophagy to degrade IL-1β, resulting in mechanisms to optimize pathogen control and yet avoid autoimmunity (116).

While relationships between epigenetic regulation, autophagy, and immunity are just now being elucidated, the study of HDAC inhibitors in cancer demonstrates some of the complexity of epigenetic manipulations. For example, studies of breast cancer carcinogenesis in the presence of the chemopreventative DNMT1 inhibitor, 3,6-dihydroxyflavone (3,6-DHF), demonstrated reduced DNA methylation with resulting activation of autophagy, as well as epigenetic activation of the *MIR21* promoter, resulting in an unexpected induction of the NOTCH-1 pathway (120). Applying some of the known pathways in Figure 1 may help to anticipate some side effects. For example, mTOR-dependent signaling of SIRT1-dependent H3K9 acetylation would be expected to increase autophagy activity (121). However, high mTOR activities would be expected to phosphorylate the S249 amino acid of DCP2, resulting in increased autophagy-related transcript suppression, which modulates the effect of autophagy on IL-1β and autoimmunity with potential reductions in pathogen clearance. However, without experimental probing of these relationships, it is difficult to discern which effects would predominate under a given condition of autoimmunity or infection. Clearly, the study of the role of epigenetic networks in autophagy and autoimmunity is in its infancy, but is critical to the application of a developing repertoire of epigenetic pharmaceuticals to autoimmunity as well as for the anticipation of their potential side effects.

## Author Contributions

PW and JH participated in writing and preparation of the manuscript.

## Conflict of Interest Statement

The authors declare that the research was conducted in the absence of any commercial or financial relationships that could be construed as a potential conflict of interest.
